# External-Compression Headache Caused by Wearing a Work Helmet Lasting for Approximately Seven Months: A Case Report

**DOI:** 10.7759/cureus.40018

**Published:** 2023-06-05

**Authors:** Yasutaka Sadamoto

**Affiliations:** 1 Neurosurgery, Headache Center, Takanoko Hospital, Matsuyama, JPN

**Keywords:** mirogabalin besylate, external compression headache, sensitization, allodynia, primary headache disorder, helmet use

## Abstract

External compression headaches are relatively rare. However, the consultation rate is low and the disease is not well recognized. This report describes a patient who developed intolerable headaches after wearing a helmet at a construction site and required approximately seven months of leave from work. The patient continued to wear the helmet even after the onset of an external compression headache, which worsened. In particular, acute drug treatment is ineffective, resulting in the need for long-term absence. Based on the discrepancy between prevalence and consultation rates, educating occupational workers and workplaces requiring helmet use for external compression headaches is essential.

## Introduction

External compression headache (ECH) is a relatively rare primary headache, with a prevalence of approximately 4% in the population, and is more prevalent in women; certain populations that specifically require headwear and helmets (e.g., construction workers and military personnel) are at a higher risk of developing this type of headache [1]. ECH was diagnosed in 82 individuals at the Military Police Hospital in Rio de Janeiro between September 2000 and March 2002 [2]. A study of 279 Danish Military personnel required to wear helmets at all times found ECH in approximately 30% of them [3]. Of the 28,383 patients who visited seven Italian tertiary headache centers between 2014 and 2017, only five had external-pressure headache (EPH), including ECH, and acknowledged deviation from prevalence [4].

The mechanism of ECH remains unknown; however, it is generally believed to result from sustained compression of pericranial soft tissues without damage to the scalp [1,5]. Continuous pressure against the head can affect localized tissue and irritate the superficial sensory nerves (trigeminal, occipital, and cervical nerve branches) innervating the face, head, and neck [1,5]. ECH is not well recognized because the headache disappears when pressure is removed and medical attention tends to be postponed [6].

We encountered the case of a construction site supervisor who always wore a helmet and developed intolerable headaches due to wearing the helmet, lasting approximately seven months. In the present case, the patient continued to wear a helmet after the onset of ECH, and the repeated pain may have caused central sensitization. Detailed case reports on ECH are rare, and we believe that this study will contribute to the understanding of the disease.

## Case presentation

The patient was a 45-year-old man with no relevant medical or family history. He had no specific illnesses, including anxiety or depression, noted during routine physical examination at work. He did not drink alcohol but smoked approximately 20 cigarettes per day, with a body mass index of 24.60 kg/m^2^. He has worked at a construction company since the age of 22 years. He wore a helmet for 6-7 h a day. The helmet he wore was a typical construction site helmet of approximately 400 mg of hard material, with a band inside that was adjustable to some extent. The helmets were regularly renewed; however, there were no major changes in the model.

On May 25, 2019, while working with a helmet without any specific trigger, the headache gradually developed on the right side. The headache was a non-pulsating, tightening pain that started in the right periorbital area and continued into the right occipital region. Headache was not accompanied by nausea, vomiting, photophobia, phonophobia, or osmophobia, and was not aggravated by walking or climbing stairs. Additionally, the headache was not accompanied by any facial autonomic symptoms, such as lacrimation, nasal discharge, or nasal obstruction. On a numerical analog scale of 1-10, the pain was of intensity 3-4, which disappeared within an hour after removing the helmet. From the onset, he endured headaches and continued to work, and after about two weeks of onset, his headache gradually became more severe, reaching 7/10 in intensity. He could wear the helmet only for 1-2 h, so he visited a private hospital in the third week after the onset of the disease, where he was treated by a headache specialist on an outpatient basis.

**Figure 1:**
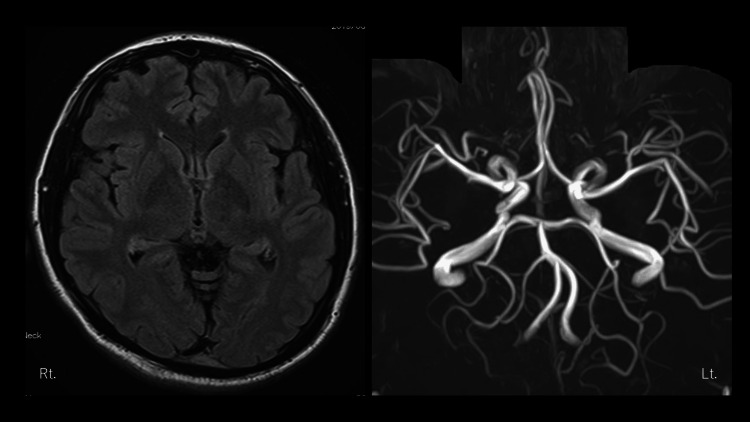
Head-plane magnetic resonance imaging and magnetic resonance angiography at initial visit. There were no apparent organic diseases.

The patient’s temperature was 36.5°C, and his weight did not change during the year prior to the visit. He had clear consciousness, stable gait, no muscle weakness in the extremities, normal deep tendon reflexes, no abnormalities in the cranial nervous system, no pyramidal or cerebellar signs, no complaints of numbness, no calm conversation, normal mental status, and a normal fundus examination. We considered it necessary to exclude secondary headaches because the patient had never experienced headaches in the past and the headaches were interfering with his work. However, we found no organic diseases on the head-plane magnetic resonance imaging (Figure 1). Blood tests showed no inflammatory reaction, and his liver and kidney function parameters and blood glucose levels were within normal ranges. We did not perform an electroencephalogram or cerebrospinal fluid examination.

We diagnosed ECH, since helmet use is related, and prescribed non-steroidal anti-inflammatory drugs (e.g., indomethacin, loxoprofen, and diclofenac potassium) for acute treatment. Acute medication did not alleviate the headache, which lightened within an hour of removing the helmet. He began to have difficulty concentrating on his work because of headaches; he sometimes went to the restroom for helmet removal. After working patiently, he experienced a headache lasting approximately 3-4/10 in intensity, even without wearing a helmet. This headache was a superficial tingling discomfort and mild numbness over the entire head. Mirogabalin besylate (20 mg/day) was prescribed, which improved the headache while the helmet was off. Excruciating headaches did not appear when the patient did not wear a helmet. However, this occurred when he tried to wear a loose hat. His workplace required him to wear a helmet by law. He consulted his workplace and took leave of absence beginning in the second month of his illness. We continued to prescribe mirogabalin besylate (5 mg/day). He had no problems wearing a loose hat from the sixth month of onset and returned to work while wearing a helmet from the seventh month onwards.

The patient quit smoking and attended the clinic every four to eight weeks for six months after ECH improvement. We discontinued mirogabalin besylate after one month of ECH improvement, but ECH did not recur.

## Discussion

ECH is a primary headache classified as external-pressure headache (EPH) in the International Classification of Headache Disorders, third edition (ICHD-3) [7]. The International Headache Society (IHS) criteria for ECH are as follows: (A) At least two episodes of headache fulfilling criteria B-D. (B) Brought on by and occurring within 1 h of sustained external compression of the forehead or scalp. (C) Maximal at the site of external compression. (D) Resolving within 1 h after external compression is relieved. (E) Not better accounted for by another ICHD-3 diagnosis.

Primary headaches that produce moderate-to-severe headaches on one side include migraine, trigeminal autonomic cephalalgia, including cluster headaches, and other primary headaches, including ECH. In this case, ECH was diagnosed based on the ICHD-3 because the headache occurred repeatedly, associated with putting on and taking off the helmet [7]. Furthermore, the patient's headache was not accompanied by symptoms, such as nausea, photophobia, or facial autonomic symptoms. The pain sensation resulting from ECH is often characterized as constant, dull, and severe pain, which hurts the most at the location of the applied pressure. In most cases, remission is observed after discontinuation of the pressure source [1].

A previous study showed that headache medications that effectively relieved their typical headaches were not effective for attacks related to head compression; during the first return consultation, after five weeks of not wearing a helmet, all patients reported a complete absence of the associated headache attacks [2]. Another study showed that the most frequently reported site of headache was the front of the head, and 20% (17/85) reported remission of their headache by continued use of the helmet [3]. In the present case, while wearing a work helmet, the patient developed an intolerable headache on the right side that did not respond to non-steroidal anti-inflammatory drugs (NSAIDs), which made it difficult for him to perform his work. Repeated painful stimuli are thought to cause central and peripheral sensitization in primary headaches [8]. In the present case, the severity of the headache gradually worsened from the onset, and mild-to-moderate headache with superficial tingling discomfort and mild numbness persisted even when the patient was not wearing a helmet. Therefore, we considered the possibility that central sensitization may have caused allodynia. In this case, the medication was ineffective, particularly for acute treatment, and headgear other than the helmet established by the company was not allowed. The patient required prolonged leave of absence to avoid pressure. However, there have been cases of relief after changing helmets [9].

In contrast, mirogabalin besylate was effective for headaches with numbness that persisted even after the removal of helmet, a condition considered to be allodynia. The International Association for the Study of Pain defined neuropathic pain as “pain caused by a lesion or disease of the somatosensory nervous system” [10]. Mirogabalin besylate, a potent and specific ligand for the α_2_δ subunit of voltage-gated calcium channels, is a gabapentinoid that was developed for the treatment of peripheral neuropathic pain in Japan. It has the distinguishing feature of persistent binding to the α_2_δ-1 subunit, which plays an important role in neuropathic pain, including allodynia [11]. Unlike previous studies, the present case required a long period of time for ECH to heal. The patient also had allodynia due to ECH, and mirogabalin besylate was effective in treating allodynia [2].

This study has some limitations. It cannot be denied that the patient was forced to take a prolonged leave of absence, which added a certain amount of social stress and may have affected his pain. In this regard, psychiatric evaluations are insufficient. The natural history of ECH remains unknown in previous studies, detailed case reports of ECH are rare, and we believe that this study will contribute to the understanding of the disease [2,3]. This case suggests that helmet-related ECH should be properly diagnosed and helmet use should be avoided or changed before peripheral and central sensitization progresses and symptoms worsen. Since the coronavirus disease 2019 (COVID-19) outbreak, there have been increasing reports of EPH and ECH in healthcare workers using personal protective equipment (PPE) such as N95 masks and goggles [5,6]. Based on the discrepancy between prevalence and consultation rates, It is essential to more research into ECH, and educate occupational workers and workplaces that require PPE and helmet use.

## Conclusions

In ECH, wearing causative helmets and headgears should be avoided for a certain period, or alternatives should be considered. Based on the discrepancy between the prevalence and consultation rates, it is essential to educate occupational workers and workplaces about ECH that require PPE and helmet use. Medical institutions should be aware of ECH.
